# Residual Stress Distribution Design for Gear Surfaces Based on Genetic Algorithm Optimization

**DOI:** 10.3390/ma14020366

**Published:** 2021-01-13

**Authors:** Zhou Chen, Yibo Jiang, Zheming Tong, Shuiguang Tong

**Affiliations:** 1 State Key Laboratory of Fluid Power and Mechatronic Systems, Zhejiang University, Hangzhou 310027, China; chenzhou92@zju.edu.cn (Z.C.); 11925084@zju.edu.cn (Y.J.); cetongsg@zju.edu.cn (S.T.); 2 School of Mechanical Engineering, Zhejiang University, Hangzhou 310027, China

**Keywords:** residual stress distribution, rolling contact fatigue, Fatemi–Socie criterion, genetic algorithm

## Abstract

The rolling contact fatigue of gear surfaces in a heavy loader gearbox is investigated under various working conditions using the critical plane-based multiaxial Fatemi–Socie criterion. The mechanism for residual stress to increase the fatigue initiation life is that the compressive residual stress has a negative normal component on the critical plane. Based on this mechanism, the genetic algorithm is used to search the optimum residual stress distribution that can maximize the fatigue initiation life for a wide range of working conditions. The optimum residual stress distribution is more effective in increasing the fatigue initiation life when the friction coefficient is larger than its critical value, above which the fatigue initiation moves from the subsurface to the surface. Finally, the effect on the fatigue initiation life when the residual stress distribution deviates from the optimum distribution is analyzed. A sound physical explanation for this effect is provided. This yields a useful guideline to design the residual stress distribution.

## 1. Introduction

The gearbox is the most important transmission system in many applications such as heavy equipment, automobiles, helicopters, wind turbines, etc. [1,2]. A key aspect in designing a gearbox is to prolong the gear tooth longevity. Rolling contact fatigue (RCF) that results from the cyclic rolling–sliding contact in the gear meshing pair is a common failure mode of the gear tooth surface [3]. It cannot be avoided and must occur as long as gears are operated long enough. RCF first leads to the initiation of fatigue microcracks. These microcracks then propagate and merge into multiple macrocracks, which can form pieces of material removed from the bulk, i.e., pitting. Pitting can accelerate tooth surface deterioration [4], cause transmission error [5], and increase vibration and noise [6], etc. Therefore, it is imperative to investigate the behavior of RCF.

Sharif et al. [7] investigated the influence of surface roughness on RCF under elastohydrodynamic (EHL) contact conditions. They pointed out that the ratio of the film thickness over the surface roughness is the key index of the fatigue performance. Li and Kahraman [8] proposed a novel model for the progression of pitting behavior in spur gear contacts. The novelty lies in the model’s capability of simultaneously considering various time-varying variables such as contact load, contact radii, and surface velocities. Paulson et al. [9] found that Hertzian pressure distribution, compared with EHL under the same contact loading, reduces fatigue life. Moreover, the material degradation affected the contact pressure distribution and somehow increased the fatigue life. Wang et al. [10,11] performed extensive finite element simulations of RCF in carburized gear surfaces and revealed that proper carburization process can improve the fatigue performance. Golmohammadi and Sadeghi [12] studied the effect of surface dents on RCF and found that a dent of sharp edge and high pile-up can significantly deteriorate the fatigue performance. 

**Figure 1:**
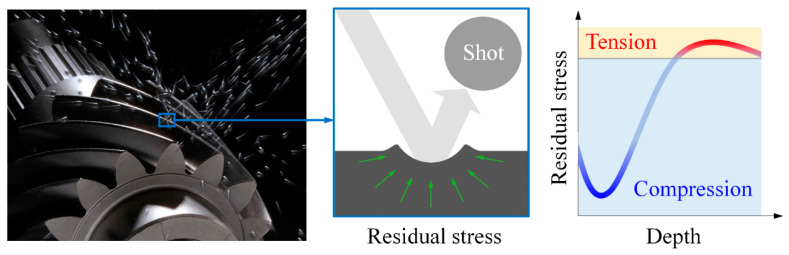
Shot peening in gear manufacturing and typical residual stress (RS) distribution.

The work in [7,8,9,10,11,12] provided useful insights into RCF. Unfortunately, they ignored the residual stress (RS) induced by shot peening, which is an essential treatment to enhance the fatigue performance in gear manufacturing (see Figure 1, where the typical RS distribution induced by shot peening is shown [13]). Liu et al. [14] revealed that compressive residual stress (CRS) can increase fatigue initiation life in contrast to tensile residual stress (TRS). Paladugu and Hyde [15] additionally considered the material microstructure under dry and lubricated conditions and confirmed the conclusion in [14]. Wang et al. [16] compared TRS and CRS of the same magnitude and found that the decrease in fatigue initiation life induced by TRS is four times the increase by CRS. Xu et al. [17] presented a model to predict the initiation and propagation of cracks due to RCF and showed that the prediction considering residual stresses is in better agreement with the experiments. Ooi et al. [18] theoretically and experimentally demonstrated that the effect of residual stress on the RCF life of carburized AISI 8620 steel varies with the percentage of restrained austenite. 

Although RS was included in the investigation of RCF in [14,15,16,17,18], the effect of the distribution of residual stress was not addressed. Guan et al. [19] and Zhang et al. [20] examined the effect of CRS distribution resulting from different shot peening processes and pointed out the potential of tailoring CRS distribution to improve the fatigue performance. However, no further study on this issue was carried out. Walvekar and Sadeghi [21] studied the effect of CRS distributions through a parametric study and demonstrated the dependence of fatigue performance on variables associated with the CRS distribution profile, such as the depth and magnitude of the peak CRS and the CRS layer thickness (see Figure 1). Morris et al. [22] included the effect of material microstructure and reported that higher CRS near the surface results in deeper initiation of fatigue cracks, which therefore enhances the fatigue performance. 

As can be seen from the above literature review, relevant works addressing the effect of RS distribution [19,20,21,22] have provided useful information for the RS distribution design. However, they only considered the effect of a single variable of the distribution profile. No efforts were made to optimize the RS distribution to obtain the best fatigue performance. Therefore, the current study adopts a genetic-algorithm-based optimization scheme to search for the optimum RS distribution. Based on the optimization results, some recommendations for the RS distribution design are provided. As an example, this work is performed for the surfaces of gears used in the gearbox of a heavy loader. 

## 2. Residual Stress Distribution

**Figure 2:**
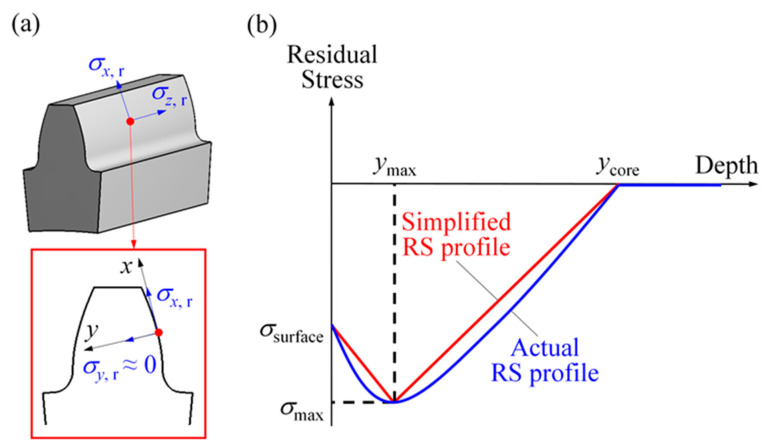
(**a**) RS components demonstrated on a gear tooth and (**b**) a simplified RS distribution profile along the depth.

The components of residual stress are depicted in Figure 2a. Both normal components in the *x* and *z* directions exhibit identical distributions, while the component in the *y* direction is small enough to be ignored [23]. The typical RS distribution along the depth (see, e.g., [13]) induced by shot peening is shown in Figure 1. However, the distribution is too complex and requires too many variables to define its profile. This disables efficient optimization of RS distribution (introduced in Section 5). Thus, as was done in [21], the distribution profile is simplified by ignoring the detrimental TRS and assuming the profile to consist of straight lines, as shown in Figure 2b. This simplification reduces the number of variables of the distribution profile without losing the basic features of the RS distribution. The design variables for the optimization of the RS distribution profile are as follows:

(1) *σ*_surface_—the RS at the surface;
(2) *σ*_max_—the peak RS;
(3) *y*_max_—the depth of *σ*_max_;
(4) *y*_core_—the depth where RS vanishes.

The values of these variables can be controlled to formulate different RS distributions by adjusting shot peening process parameters such as the shot velocity, shot size, coverage, etc. [24]. 

## 3. Gear Contact Model

**Figure 3:**
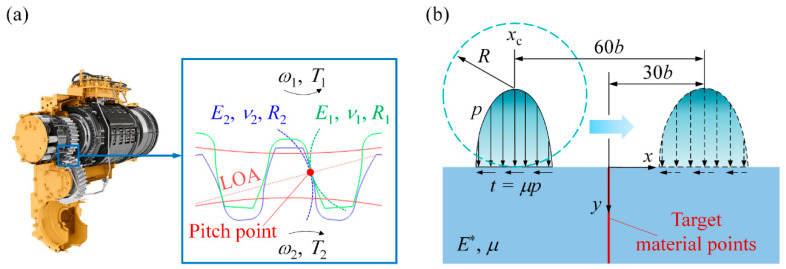
(**a**) The meshing gear pair in a heavy loader gearbox and (**b**) the simplified contact model.

Figure 3a presents the contact problem in a pair of meshing gears used in the 157 kW gearbox of a heavy loader. The geometry and material properties of the gears are presented in Table 1. The contact of a helical gear pair is equivalent to that of a tapered roller and a deformable half-space, to which the solution can be found in [25]. The present study focuses on the methodology for the design of RS distribution. The methodology itself is universal and valid for any type of gear. Therefore, for the convince of demonstrating this methodology, the helix angle is not considered and both the pinion and gear are regarded as spur gears, the simplest type of gear. The contact of meshing teeth is equivalent to that of two cylinders of varying radii (indicated by two dashed arcs), which depends on the position of the meshing point on the line of action (LOA). Since the gear contact is elastic, the contact of two cylinders can be simplified as that of a rigid cylinder of equivalent radius *R* and a deformable half-space of equivalent Young’s modulus E^*^ in plane strain condition [23], as shown in Figure 3b.

(1) $$\frac{1}{R}=\frac{1}{R_{1}}+\frac{1}{R_{2}}$$

(2) $$\frac{1}{E^{*}}=\frac{1-\nu_{1}^{2}}{E_{1}}+\frac{1-\nu_{2}^{2}}{E_{2}}$$

The contact model in Figure 3b aims to simulate the contact in the region close to the pitch point, where it is more susceptible to RCF since there is only one pair of teeth in contact [26]. Thus, the rated meshing force per unit length *P*_rated_ = 423 N/mm, which can be easily obtained using the parameters in Table 1 [27], is the normal contact load. To gain basic physical insights, the variation of *R* is ignored and *R* = 15.231 mm at the pitch point is used for the rigid cylinder. Due to the involute geometry, the gear surface is subjected to rolling–sliding contact. This type of contact loading can be represented by Hertzian pressure with shear traction:

(3) $$p=\left\{ \begin{array}{c} \sqrt{\frac{PE^{*}}{\pi RL}\left(1-{\left(\frac{x-x_{c}}{b}\right)}^{2}\right)},\left|x-x_{c}\right|\leq b\\ 0,\left|x-x_{c}\right|>b \end{array}\right.$$

(4) t=μp

where *p* is the normal pressure, *P* is the applied normal load, *x*_c_ is the x-coordinate of the normal pressure center, *b* = (4*PR*/(πE^*^))^0.5^ is the half contact width, *t* is the shear traction, and *μ* is the friction coefficient ranging from 0 to 0.3, representing ideally lubricated to dry friction conditions.

The analytical solution for the stress/strain field in the half-space was provided in [28] (see Appendix A). When the RS distribution is incorporated in the half-space, it can be demonstrated that the deformation in the half-space is still elastic and the RS distribution is not altered as the number of loading cycles increases. Therefore, the RS distribution can be directly added to the stress solution provided in [28] to obtain the stress field with the presence of RS. Since material points at the same depth are subjected to the same loading cycle, the fatigue analysis will be conducted only for target points at *x* = 0 (see the red line in Figure 3b). *x*_c_ is varied from −30*b* to 30*b* so that the pressure profile moves over a sufficiently long distance to ensure that the material points at *x* = 0 undergo a complete loading cycle.

## 4. Fatemi–Socie Multiaxial Fatigue Criterion

The complex multiaxial stress state exists in the half-space subjected to rolling–sliding contact. Moreover, this kind of contact loading is a typical non-proportional loading that results in stress components varying non-proportionally during the loading process, i.e., the principal directions constantly change. To analyze the RCF, a proper multiaxial fatigue criterion that takes the effect of the loading non-proportionality into account is needed. The critical plane-based Fatemi–Socie fatigue criterion [29] showed good agreement between the predicted and experimental results in RCF in gears [30] and was adopted extensively in many theoretical studies on gear RCF (see, e.g., [14]). Therefore, this criterion is also used in the present study.

The Fatemi–Socie fatigue criterion states that a fatigue microcrack at a material point is initiated along the critical plane that has the maximum shear strain range over a loading cycle. However, each material point usually has multiple critical planes. In this case, the fatigue microcrack occurs along the critical plane with the maximum fatigue damage. The fatigue damage of one material point on an arbitrary plane can be quantified by the Fatemi–Socie damage parameter (FSDP) as follows:

(5) $$\mathrm{FSDP}=\frac{\Delta \gamma }{2}\left(1+k\frac{\sigma_{n,\max}}{Y}\right)$$

where Δ*γ* and *σ*_n,max_ are the maximum shear strain range and maximum normal stress on this plane over one loading cycle, respectively; *Y* is the material yield strength, and *k* is a fitting parameter ranging from 0 to 1 [31]. For high cycle fatigue, which is the case in the present study, *k* approaches unity. Thus, *k* = 1 is adopted. The normal stress component is included to consider the fact that compressive stress tends to retard fatigue microcrack initiation while tensile stress accelerates the initiation. The maximum FSDP on all the critical planes is denoted by *D*_FS_. The fatigue initiation life *N*_f_ of a material point is related to *D*_FS_ by:

(6) $$D_{\mathrm{FS}}=\frac{\tau_{f}^{\prime }}{G}{\left(2N_{f}\right)}^{m}+\gamma_{f}^{\prime }\;{\left(2N_{f}\right)}^{n}$$

where *N*_f_ is the number of loading cycles when the fatigue microcrack is initiated; *G* = *E*/(2 + 2*ν*); $\tau_{f}^{\prime }$ and $\gamma_{f}^{\prime }$ are the shear fatigue strength coefficient and the shear fatigue ductility coefficient, respectively; *m* and *n* are the fatigue strength exponent and the fatigue ductility exponent, respectively. $\tau_{f}^{\prime }$ = 1296 MPa, $\gamma_{f}^{\prime }$ = 0.437, *m* = −0.087, and *n* = −0.58. Note that temperature may have an impact on the above parameters. However, this is not considered since this paper only focuses on demonstrating the methodology for the design of RS distribution. One can easily incorporate a temperature-dependent fatigue citation in the proposed methodology to obtain a temperature-dependent optimum RS distribution.

**Figure 4:**
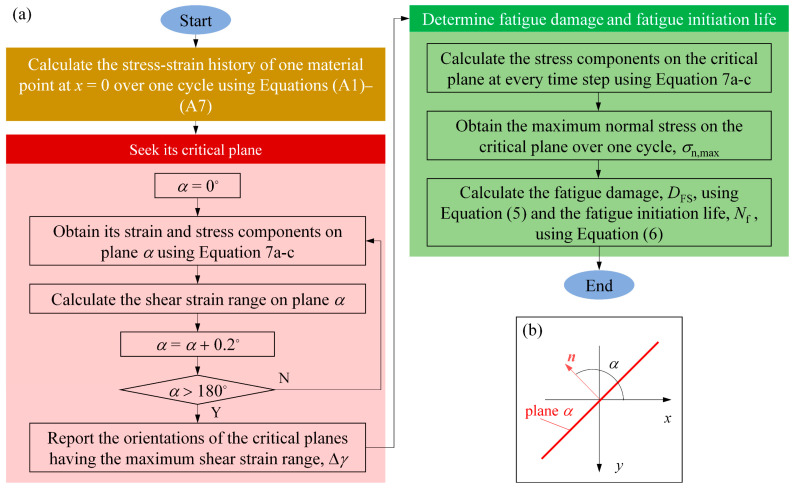
(**a**) The numerical procedure to determine *D*_FS_ and *N*_f_ of a material point and (**b**) the definition of plane orientation *α*.

Figure 4a shows a flow chart that illustrates the procedure to predict *D*_FS_ and *N*_f_ for one material point. The critical plane needs to be first identified. To this end, the stress–strain history over a loading cycle for a material point should be obtained. This can be done by taking the coordinates of this point into Equations (A1)–(A7) and varying *x*_c_ from −30*b* to 30*b*. Then, all the candidate planes that pass this point are examined to find the critical plane. Each candidate plane is characterized by the angle *α* between its normal direction and the *x*-axis (see Figure 4b), ranging from 0° to 180° with intervals of 0.2°. The stress–strain components on each candidate plane can be obtained by coordinate transformation.

(7a) $$\varepsilon^{\prime }=M\varepsilon M^{T},\;\sigma^{\prime }=M\sigma M^{T}$$

(7b) $$\varepsilon =\left[\begin{array}{ccc} \varepsilon_{xx} & \gamma_{xy}/2 & 0\\ \gamma_{yx}/2 & \varepsilon_{yy} & 0\\ 0 & 0 & \varepsilon_{zz} \end{array}\right],\;\sigma =\left[\begin{array}{ccc} \sigma_{xx} & \sigma_{xy} & 0\\ \sigma_{yx} & \sigma_{yy} & 0\\ 0 & 0 & \sigma_{zz} \end{array}\right]$$

(7c) $$M=\left[\begin{array}{ccc} \cos\alpha & \sin\alpha & 0\\ -\sin\alpha & \cos\alpha & 0\\ 0 & 0 & 1 \end{array}\right]$$

Each candidate plane’s Δ*γ* and *σ*_n,max_ can thus be obtained. Then, the critical planes can be determined. *D*_FS_ is the largest FSDP among all the critical planes. Finally, the fatigue initiation life of this point, *N*_f_, can be calculated using Equation (6). 

## 5. Optimization Scheme

The genetic algorithm (GA) is an optimization methodology inspired by the “survival of the fittest” rule in the theory of evolution. It is an efficient and robust method to solve complex optimization problems [32,33,34,35]. In gear-related problems, it has been used to optimize the tooth profile [35], maintain the reliability of gear transmission [36], and improve the load-sharing performance of planetary gears [37]. In this paper, the GA is used to search for the optimum RS distribution that maximizes the fatigue initiation life of the gear surface, which is (*N*_f_)_min_, i.e., the minimum *N*_f_ among all the points. Since (*N*_f_)_min_ increases with decreasing (*D*_FS_)_max_ according to Equation (6), the objective of this optimization problem is to minimize (*D*_FS_)_max_. As can be seen from Figure 2, *σ*_surface_, *σ*_max_, *y*_max_, and *y*_core_ are the four design variables for the optimization, and thus, the constraints are applied on these four variables. 

The mathematical description of this optimization problem is as follows:

(8a) $$\underset{x\in {\mathbb{R}}^{4}}{\min}\;\;{\left(D_{\mathrm{FS}}\right)}_{\max}\;\;\left(x\right)$$

(8b) $$s.t.\;\;\sigma_{\mathrm{surface}}>\sigma_{\max}$$

(8c) $$y_{\mathrm{core}}>y_{\max}$$

(8d) $$100\;\mathrm{MPa}<\sigma_{\mathrm{surface}}-\sigma_{\max}<600\;\mathrm{MPa}$$

(8e) $$\frac{1}{3}\left(y_{\mathrm{core}}-y_{\max}\right)<y_{\max}<y_{\mathrm{core}}-y_{\max}$$

(8f) $$-1000\;\mathrm{MPa}\;<\sigma_{\max},\sigma_{\mathrm{surface}}<0$$

(8g) $$0\;<y_{\max},y_{\mathrm{core}}<1\;\mathrm{mm}$$

where *x* = (*σ*_surface_, *σ*_max_, *y*_max_, *y*_core_) is called an individual in GA. Each component of the individual is called a “gene”. The constraints in Equation (8b–g) are summarized from the existing experimental [38,39,40,41] and theoretical [13,42] results for RS distribution profiles produced using the shot peening process. 

**Figure 5:**
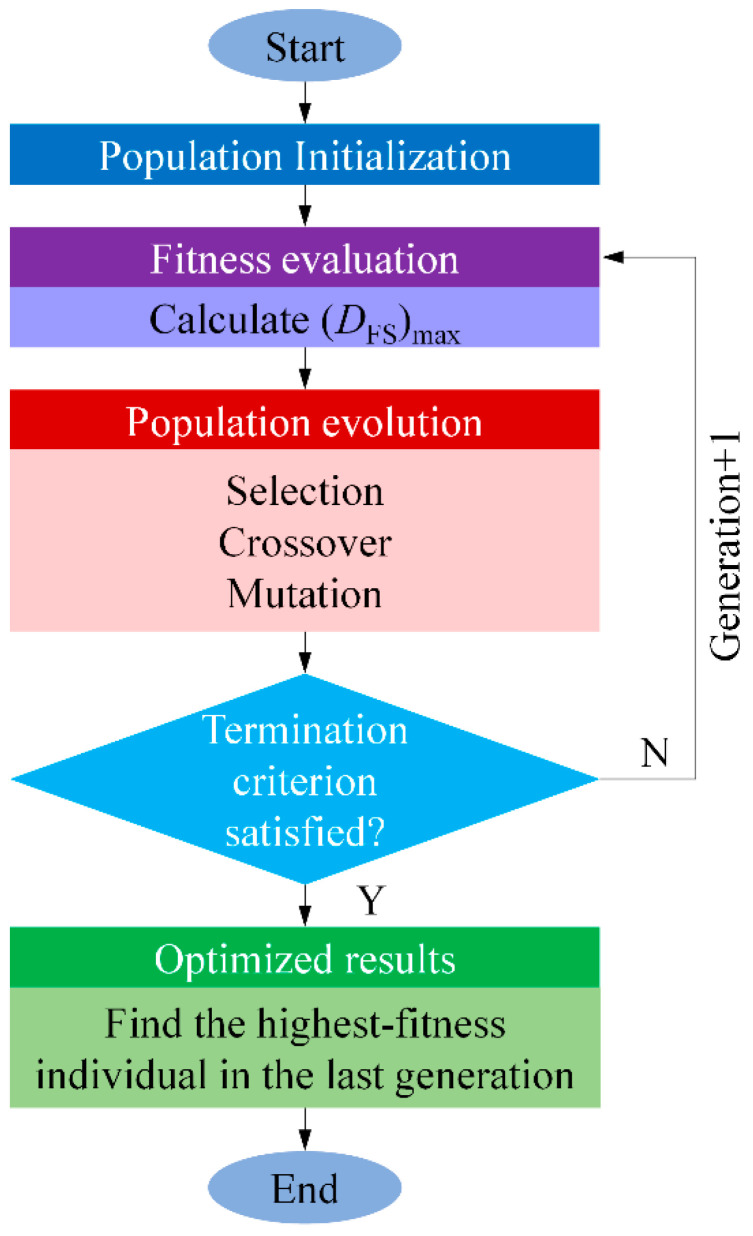
Basic steps in the genetic algorithm (GA).

The basic steps in the GA [43] are illustrated by the flow chart in Figure 5. 

Step 1: Population initialization. Generate a random initial population containing *N* individuals that satisfy the constraints in Equation (8b–g). 

Step 2: Fitness evaluation. Calculate (*D*_FS_)_max_ for each individual and use 1/(*D*_FS_)_max_ to measure its fitness. Individuals having higher fitness are more likely to survive.

Step 3: Population evolution. Three genetic operators are needed for population evolution to select and generate individuals with high fitness and eliminate those with low fitness. The selection operator is first applied to specify individuals of higher fitness as parents for the next generation. Then, two parents exchange one or multiple genes and generate two new individuals using the crossover operator. The crossover fraction, *P*_c_, decides how many individuals are generated in the next generation. Finally, genes of individuals are altered randomly using the mutation operator. 

Step 4: Termination. The optimization process is terminated once one of the following two criteria is satisfied:

(1) The generation number reaches the prescribed upper limit *G*_max_;
(2) The relative change in the highest fitness over *G*_s_ generations is less than the function tolerance value *e*.

The optimum solution is the individual with the highest fitness in the last generation. The input parameters for the GA are listed in Table 2.

## 6. Results and Discussion

### 6.1. Mechanism for RS to Increase (N_f_)_min_

**Figure 6:**
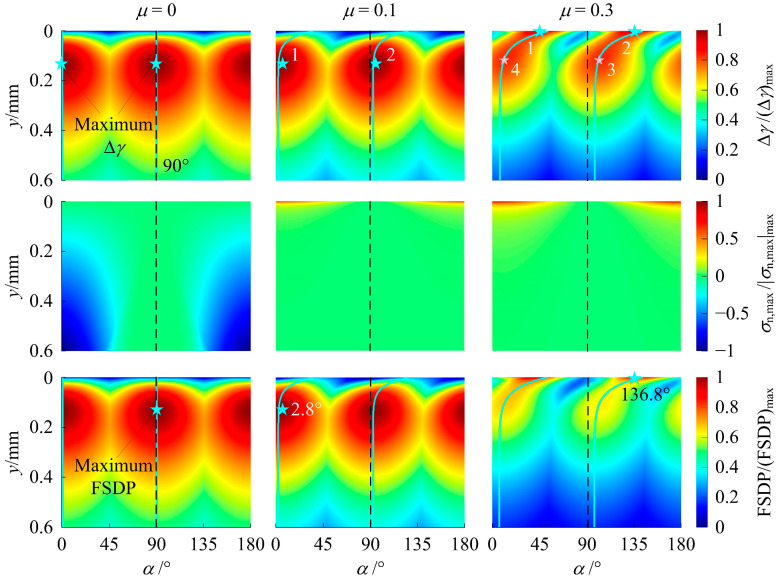
The contour of Δ*γ*/Δ*γ*_max_, *σ*_n,max_/|*σ*_n,max_|_max_ and the Fatemi–Socie damage parameter (FSDP)/(FSDP)_max_ for *P*/*P*_rated_ = 1 and *μ* = 0, 0.1, and 0.3.

As mentioned, since the fatigue damage along the *x*-axis direction exhibits a uniform distribution, fatigue analysis was performed for material points at *x* = 0. Figure 6 shows the shear strain range Δ*γ*, the maximum normal stress over a loading cycle *σ*_n,max_, and the FSDP on all the candidate planes (0° ≤ *α* ≤ 180°) of material points at *x* = 0 for three working conditions where *P*/*P*_rated_ = 1 with *μ* = 0, 0.1, and 0.3 without the presence of RS. These results are normalized by their maximum absolute values. The critical planes of each material point along the depth are denoted by the solid cyan lines in the Δ*γ* and FSDP distribution.

As would be expected from the theory of elasticity [44], each point has two critical planes with equal maximum Δ*γ*. These two planes have an *α* with a difference of 90°. For *μ* = 0, the critical plane orientations are independent of the depth. For *μ* ≠ 0, they depend on the depth due to the shear traction induced by friction. The effect of shear traction decays with increasing depth. The effect of *σ*_n,max_ on the fatigue damage is demonstrated by the difference between the distributions of Δ*γ* and FSDP. *σ*_n,max_ is compressive for *μ* = 0 and tensile for *μ* ≠ 0. For *μ* = 0 and 0.1, the effect of *σ*_n,max_ is negligible. Due to the mild friction on the surface, the maximum damage occurs below the surface. On the other hand, for *μ* = 0.3, the effect of *σ*_n,max_ is appreciable and the high friction gives rise to the maximum damage appearing on the surface. For all the three cases (*P*/*P*_rated_ = 1; *μ* = 0, 0.1, and 0.3), since the *σ*_n,max_ distribution over *α* is not uniform, the FSDP on the two critical planes is different according to Equation (5). Thus, there is only one critical plane having the largest FSDP (i.e., *D*_FS_) and the orientation of the critical plane corresponding to *D*_FS_ is denoted by *α*_c_. 

**Figure 7:**
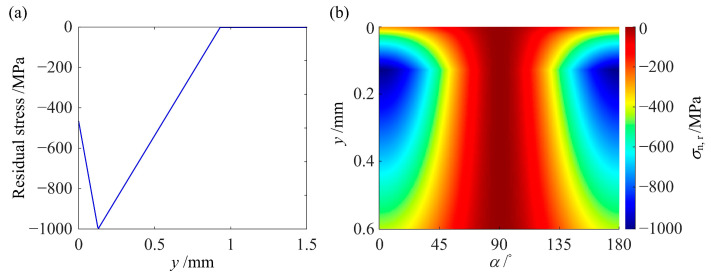
(**a**) A typical RS distribution profile and (**b**) the contour of *σ*_n,r_ resulting from this RS distribution.

Figure 7 shows a typical RS distribution and the normal component *σ*_n,r_ introduced by this RS distribution on all candidate planes. The *σ*_n,r_ distribution in Figure 7b can be added to the *σ*_n,max_ distribution without RS in Figure 6 to obtain the new *σ*_n,max_ distribution with RS. In this way, the FSDP distribution can be changed by RS. Nonetheless, there is an exception that when *α*_c_ is close to 90° (see Figure 6, *μ* = 0), *N*_f_ will remain unchanged since RS will have zero normal components on the *α*_c_ plane and will not be able to decrease *D*_FS_ in such a case. 

**Figure 8:**
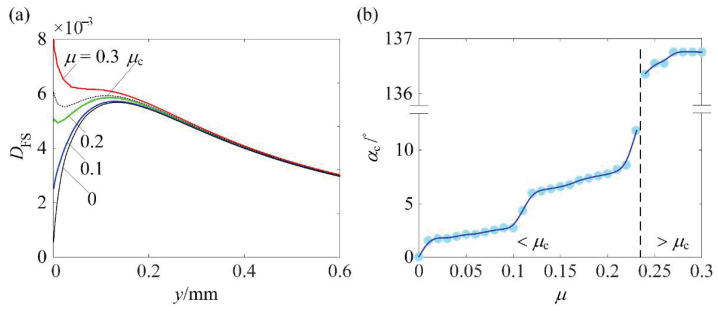
(**a**) *D*_FS_ distribution along the depth for *P*/*P*_rated_ = 1, *μ* = 0.1, 0.2, and 0.3, and (**b**) *α*_c_ as a function of *μ*.

Figure 8a presents the *D*_FS_ distributions along the depth for different *μ* under the rated normal load. It is of interest to decrease (*D*_FS_)_max_ so that (*N*_f_)_min_ can be increased. From Figure 8a, as *μ* increases, the location of (*D*_FS_)_max_ moves from the depth of around 0.13 mm to the surface. *μ*_c_ can be defined as the critical friction coefficient. For *μ* < *μ*_c_, the location of (*D*_FS_)_max_ is below the surface. For *μ* > *μ*_c_, the location of (*D*_FS_)_max_ is on the surface. It should be noted that the *D*_FS_ distributions for different normal loadings ranging from 0.5*P*_rated_ to 1.5*P*_rated_ share similar profiles. For this reason, Figure 8a is only shown for a fixed normal load with different *μ* and *μ*_c_ = 0.23 is independent of normal loading.

Figure 8b presents *α*_c_ associated with (*D*_FS_)_max_ as a function of *μ* under the rated normal load. For 0 < *μ* ≤ 0.3, *α*_c_ associated with (*D*_FS_)_max_ is not close to 90°. This provides the solid theoretical basis that (*D*_FS_)_max_ can be decreased or (*N*_f_)_min_ can be increased by introducing a proper RS distribution. Since there are four variables to define the RS distribution profile, the optimum RS distribution that can maximize (*N*_f_)_min_ was sought using the optimization scheme introduced in the previous section.

### 6.2. Increase in (N_f_)_min_ Induced by the Optimum RS Distribution

**Figure 9:**
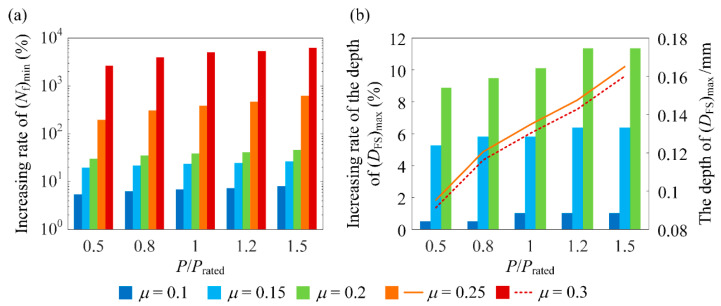
(**a**) The percentage increase in (*N*_f_)_min_, and (**b**) the percentage increase in the depth of (*D*_FS_)_max_ for *μ* = 0.1, 0.15, and 0.2 (left vertical axis) and the depth of (*D*_FS_)_max_ for *μ* = 0.25 and 0.3 (right vertical axis) when the optimum RS distribution is introduced compared to the case without RS.

Figure 9a presents the percentage increase in (*N*_f_)_min_ induced by the optimum RS distribution compared to the case without RS. From the discussion on Figure 8, the *D*_FS_ distribution depends on the working condition and so does the optimum RS distribution. Thus, the percentage increase in (*N*_f_)_min_ varies with the working condition. As the normal loading and *μ* increase, the percentage increase in (*N*_f_)_min_ increases. Compared with the increasing normal loading, *μ* has a higher impact on the percentage increase in (*N*_f_)_min_. Moreover, when *μ* > *μ*_c_, the percentage increase in (*N*_f_)_min_ is dramatically higher than that when *μ* < *μ*_c_.

Figure 9b presents the percentage increase in the depth of (*D*_FS_)_max_ induced by the optimum RS distribution for *μ* < *μ*_c_. For *μ* > *μ*_c_, (*D*_FS_)_max_ moves from the surface to the subsurface. In this case, the depth of (*D*_FS_)_max_ when the optimum RS distribution is applied is presented. The increase in the depth of (*D*_FS_)_max_ is beneficial in increasing the propagation life since more loading cycles are needed for the fatigue microcrack to propagate to the surface before a pitting particle is formed [14]. Provided that the relation between the depth of (*D*_FS_)_max_ and the propagation life is quantified, the RS distribution can be optimized to maximize the sum of the initiation life, (*N*_f_)_min_, and propagation life. Nonetheless, this is out of the scope of the present study.

Figure 9a demonstrates that the optimum RS distribution, which is obtained using the GA, can indeed substantially increase (*N*_f_)_min_. Theoretically speaking, the shot peening process parameters can be adjusted to achieve the optimum RS distribution. However, in engineering practice, it is almost impossible to precisely achieve the optimum RS distribution in this way. Thus, it is desired that the optimum RS distribution can be varied to some extent while (*N*_f_)_min_ is not sacrificed too much. By this means, it is more apt to adjust the shot peening process parameters to obtain an acceptable RS distribution and achieve a satisfying (*N*_f_)_min_. To this end, it is necessary to investigate how the variables of the RS distribution profile affect (*N*_f_)_min_ when they deviate from those of the optimum distribution. 

### 6.3. Effect on (N_f_)_min_ When the RS Distribution Deviates from the Optimum 

**Figure 10:**
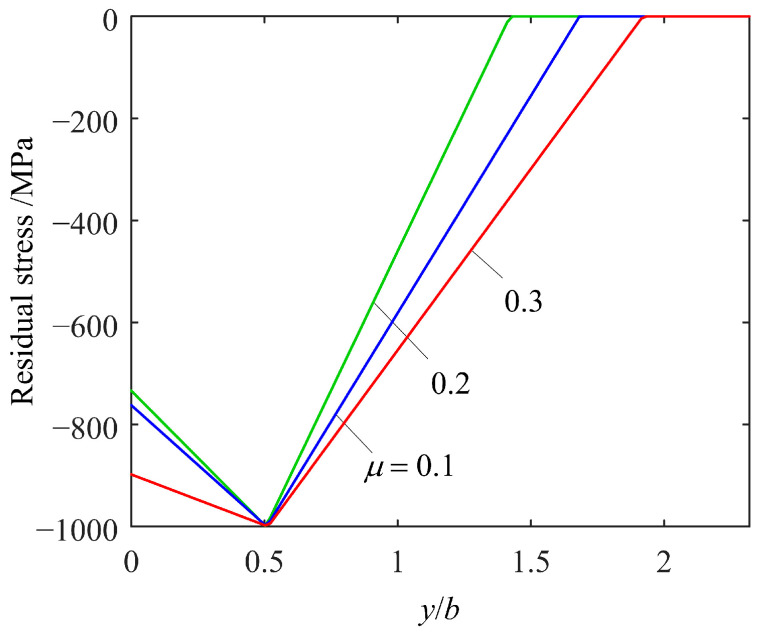
The optimum RS distribution along the depth for *P*/*P*_rated_ = 1 and *μ* = 0.1, 0.2, and 0.3.

Since the optimum RS distribution depends on the *D*_FS_ distribution, which has a weak dependence on the normal load *P*, the effect on (*N*_f_)_min_ when the RS distribution deviates from the optimum is also weakly dependent on *P*. In light of this, the optimum RS distributions under investigation are those for fixed *P* with different *μ*. Figure 10 shows the optimum RS stress distribution for *P*/*P*_rated_ = 1 and *μ* = 0.1, 0.2, and 0.3. The optimum distributions exhibit two common features. They all have nearly the same (*y*_max_)_opt_ of 0.5*b*, which is the depth of the maximum Δ*γ* [21], and the same (*σ*_max_)_opt_ = −1000 MPa, which is the lower limit of *σ*_max_. (*σ*_surface_)_opt_ and (*y*_core_)_opt_ do not exhibit a simple dependence on *μ* since they do not monotonically increase or decrease with *μ.* The effect on (*N*_f_)_min_ when the RS distribution deviates from the optimum shall be investigated by deviating a single component of (*σ*_surface_, *σ*_max_, *y*_max_, and *y*_core_) from its corresponding optimum value.

**Figure 11:**
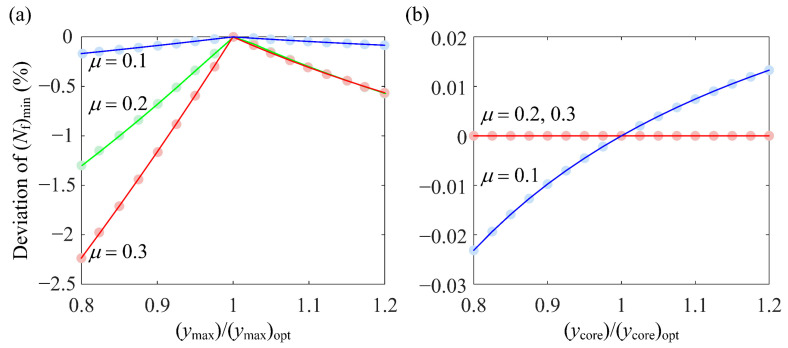
The percentage decrease in (*N*_f_)_min_ when (**a**) *y*_max_ and (**b**) *y*_core_ deviate from their optimum values for *P*/*P*_rated_ = 1 and *μ* = 0.1, 0.2, and 0.3.

**Figure 12:**
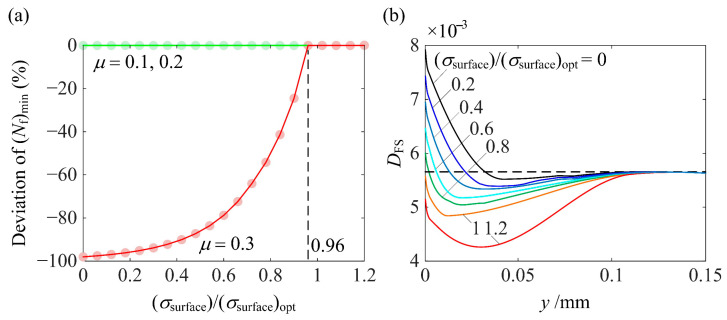
(**a**) The percentage decrease in (*N*_f_)_min_ for *P*/*P*_rated_ = 1 and *μ* = 0.1, 0.2, and 0.3 and (**b**) the variation of the *D*_FS_ distribution along the depth for *P*/*P*_rated_ = 1 and *μ* = 0.3 when *σ*_surface_ deviates from (*σ*_surface_)_opt_.

Figure 11 shows the percentage decrease in (*N*_f_)_min_ when *y*_max_ and *y*_core_ deviate from their optimum values. As can be seen from Figure 11a, the deviation from (*y*_max_)_opt_ causes a slight decrease in (*N*_f_)_min_. As *μ* increases, (*N*_f_)_min_ becomes more sensitive to the deviation of *y*_max_. From Figure 11b, it can be seen that the deviation of *y*_core_ almost has no effect on (*N*_f_)_min_. Figure 12 shows and explains the effect on (*N*_f_)_min_ when *σ*_surface_ deviates from (*σ*_surface_)_opt_. As can be seen from Figure 12a, for relatively low *μ*, deviation of *σ*_surface_ does not affect (*N*_f_)_min_ since (*D*_FS_)_max_ occurs in the subsurface. However, for *μ* = 0.3, (*N*_f_)_min_ is very sensitive to the deviation of *σ*_surface_ unless *σ*_surface_/(*σ*_surface_)_opt_ becomes larger than 0.96. This can be explained by Figure 12b. As |*σ*_surface_| increases, (*D*_FS_)_max_, which appears on the surface, decreases and the location of (*D*_FS_)_max_ moves from the surface to the subsurface when *σ*_surface_/(*σ*_surface_)_opt_ reaches 0.96. Thereafter, (*D*_FS_)_max_ in the subsurface is not affected by *σ*_surface_ and neither is (*N*_f_)_min_.

**Figure 13:**
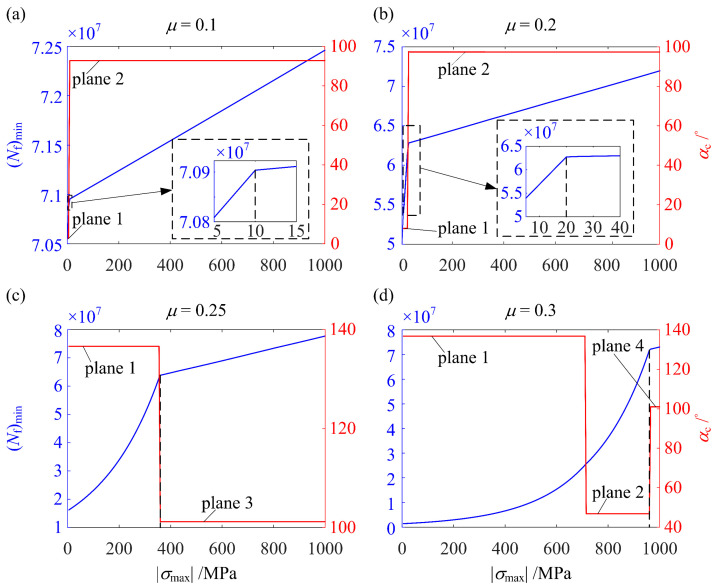
The effect on (*N*_f_)_min_ and *α*_c_ when *σ*_max_ deviates from (*σ*_max_)_opt_ = −1000 MPa for *P*/*P*_rated_ = 1 and (**a**) *μ* = 0.1, (**b**) *μ* = 0.2, (**c**) *μ* = 0.25, and (**d**) *μ* = 0.3.

As shown in Figure 10, (*σ*_max_)_opt_ for all the three cases reached its lower limit, −1000 MPa. To investigate the effect on (*N*_f_)_min_ when *σ*_max_ deviates from (*σ*_max_)_opt_, *σ*_max_ has to be increased. However, since the constraint in Equation (8b) must be satisfied, increasing *σ*_max_ should be coupled with increasing *σ*_surface_. To this end, we simply increased both *σ*_max_ and *σ*_surface_ by the same amount from (*σ*_max_)_opt_ and (*σ*_surface_)_opt_, respectively. In this way, the effect of deviation of *σ*_max_ was studied. Figure 13 shows (*N*_f_)_min_ and the orientation of the critical plane corresponding to (*D*_FS_)_max_, *α*_c_, as functions of |*σ*_max_| for four values of *μ*. The effect of deviation of *σ*_max_ on (*N*_f_)_min_ is salient in agreement with the experimental results reported in [45,46,47] that the peak RS plays a major role in the fatigue performance. 

For *μ* < *μ*_c_, the location of (*D*_FS_)_max_ remains in the subsurface. As shown in Figure 6 (*μ* = 0.1), the material point at this location has two critical planes of maximum Δ*γ*, which are denoted as planes 1 and 2 with orientations of *α*_1_ and *α*_2_ close to 0° and 90°, respectively. Initially, without RS, i.e., |*σ*_max_| = 0, plane 1 has a higher *σ*_n,max_ than plane 2 (see Figure 6). According to Equations (5) and (6), the FSDP on plane 1 is larger and is thus equal to (*D*_FS_)_max_. Consequently, *α*_c_ = *α.* Since the magnitude of the negative normal component of RS on plane 1 is larger than on plane 2 (see Figure 7b), as |*σ*_max_| increases, the FSDP on plane 1 decreases faster and is exceeded by the FSDP on plane 2. As a result, the FSDP on plane 2 becomes (*D*_FS_)_max_ so that *α*_c_ switches to *α*_2_. This results in the two distinct stages of (*N*_f_)_min_ vs. |*σ*_max_| in Figure 13a,b. 

For *μ* ≥ *μ*_c_, the location of (*D*_FS_)_max_ moves from the surface to the subsurface as |*σ*_max_| increases. As shown in Figure 6 (*μ* = 0.3), when (*D*_FS_)_max_ is on the surface, the two critical planes of maximum Δ*γ* of the material point at the location of (*D*_FS_)_max_ are denoted as planes 1 and 2. When (*D*_FS_)_max_ moves to the subsurface, the counterparts of planes 1 and 2 are denoted by planes 3 and 4. As can be seen, since *α*_1_ ≈ 45° and *α*_2_ ≈ 135°, the negative normal component on planes 1 and 2 are almost the same (see Figure 7b). This results in almost identical FSDP vs. |*σ*_max_| for these two planes. Thus, unlike Figure 13a,b, the switch of *α*_c_ from *α*_2_ to *α*_1_ (see Figure 13d) is not reflected as the deflection on the curve of (*N*_f_)_min_ vs. |*σ*_max_|. Therefore, the two distinct stages of (*N*_f_)_min_ vs. |*σ*_max_| in Figure 13c,d result from the change in the location of (*D*_FS_)_max_, which is different from the case of *μ* < *μ*_c_ in Figure 13a,b. 

**Figure 14:**
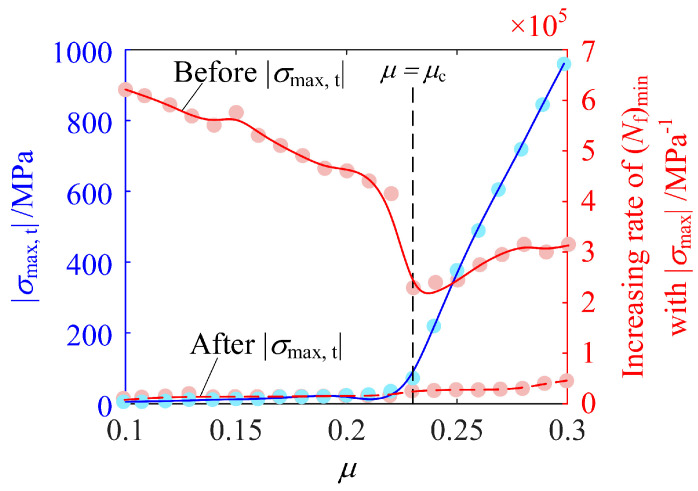
|*σ*_max,t_| and the increasing rate of (*N*_f_)_min_ with |*σ*_max_| before and after |*σ*_max,t_| as functions of *μ*.

Despite the reason leading to the two distinct stages of (*N*_f_)_min_ vs. |*σ*_max_| being different for *μ* < *μ*_c_ and *μ* ≥ *μ*_c_, it is interesting to investigate |*σ*_max,t_|, at which the two stages are divided. As demonstrated in Figure 14, |*σ*_max,t_| increases with *μ* significantly faster when *μ* > *μ*_c_. The increasing rates of (*N*_f_)_min_ with |*σ*_max_| before and after |*σ*_max,t_| are also presented in this figure. For *μ* < *μ*_c_, (*N*_f_)_min_ vs. |*σ*_max_| is bilinear and the increasing rate is thus the slope. For *μ* ≥ *μ*_c_, (*N*_f_)_min_ vs. *σ*_max_ is nonlinear when |*σ*_max_| < |*σ*_max,t_|. Thus, the increasing rate is obtained as the average of ∂(*N*_f_)_min_/∂|*σ*_max_| over 0 < |*σ*_max_| < |*σ*_max,t_| for qualitative analysis. In general, the increasing rate of (*N*_f_)_min_ with |*σ*_max_| when |*σ*_max_| < |*σ*_max,t_| is higher. This indicates that a further increase in |*σ*_max_| over |*σ*_max,t_| becomes less effective in increasing (*N*_f_)_min_. This theoretical finding is in accordance with experimental results showing that RS induced by additional shot peening is less effective in preventing fatigue in 17NiVrMo6-4 steel [48,49] and Austempered ductile iron [50]. 

The results presented in Figure 13 and Figure 14 provide useful information on the effect on (*N*_f_)_min_ when *σ*_max_ deviates from (*σ*_max_)_opt_. It is true that the ideal scenario is that |*σ*_max_| = |(*σ*_max_)_opt_| = 1000 MPa. However, a higher |*σ*_max_| requires higher shot velocity that leads to higher surface roughness. Moreover, when |*σ*_max_| > |*σ*_max,t_|, it becomes less effective in increasing (*N*_f_)_min_ by increasing |*σ*_max_|. This is especially problematic for the case of low *μ*, where *α*_c_ is close to 90° (see Figure 13a). In this case, the negative component of RS on this critical plane is trivial and the increasing rate of (*N*_f_)_min_ with |*σ*_max_| is also very slow. Therefore, when one tries to increase (*N*_f_)_min_ by increasing |*σ*_max_|, he may need to comprehensively consider the slowing increasing rate of (*N*_f_)_min_ with |*σ*_max_| and the increasing surface roughness.

## 7. Conclusions

An RCF analysis based on the multiaxial Fatemi–Socie fatigue criterion was conducted for gear surfaces in a heavy loader gearbox under various working conditions with different combinations of meshing force and friction coefficient. The effect of RS on the fatigue performance was intensively studied. As *μ* increases, the fatigue initiation location moves from the subsurface to the surface. The *μ* at which this transition occurs was defined as *μ*_c_. The mechanism for RS to increase the fatigue initiation life is that the compressive RS has a negative component on the critical plane and, thus, suppresses the fatigue damage. 

The RS distribution, simplified as a piecewise bilinear function of the depth to the surface, is described by four variables, including *y*_surface_, *y*_core_, *σ*_max_, and *σ*_surface_. The optimum RS distribution that can increase the fatigue initiation life of the gear surface (*N*_f_)_min_ to the maximum was sought using the GA. The optimum RS distribution is not universal but depends on the working condition. It was demonstrated that the optimum RS distribution can substantially increase (*N*_f_)_min_ under a wide range of working conditions. This improvement in fatigue performance is more significant for higher *μ*. Another unexpected benefit brought by the optimum RS distribution is that the fatigue initiation occurs at a deeper depth, which increases the fatigue propagation life. 

In practice, it is difficult to exactly achieve the optimum RS distribution by adjusting shot peening process parameters. Thus, it is important to explore to what extent modifications can be made on the optimum distribution without too much sacrificing of (*N*_f_)_min_. It was found that for any *μ*, be it larger or smaller than *μ*_c_, the deviation of *y*_core_ and *y*_max_ from their optimum values has a negligible effect on (*N*_f_)_min_. On the other hand, *σ*_max_ has a salient effect and |*σ*_max_| should be as large as possible. However, in practice, a cautious decision should be made since increasing |*σ*_max_| increases surface roughness and can have a trivial effect on increasing (*N*_f_)_min_ when *μ* is low or |*σ*_max_| > |*σ*_max,t_|. The effect of the deviation of *σ*_surface_ depends on *μ*. For *μ* < *μ*_c_, *σ*_surface_ has no effect. For *μ* > *μ*_c_, *σ*_surface_ should be close to (*σ*_surface_)_opt_, leaving little room for acceptable deviation of *σ*_surface_. This information can serve as a general guidance on RS distribution design by properly modifying the optimum one obtained from GA optimization.

## Figures and Tables

**Table 1:** Gear pair parameters.

Parameter	Pinion	Gear
Normal module *m*_n_/mm	5.645	
Pressure angle *α*/◦	15	
Helix angle *β*/◦	22.3	
Contact ratio *ε_α_*	1.746	
Face width *L*/mm	54	
Tooth number	*z*_1_ = 37	*z*_2_ = 48
Radius at the pitch point/mm	*R*_1_ = 27.029	*R*_2_ = 35.065
Rated output torque *T*_rated_/(N·m)	3000	
Rotating speed *n*/(r/min)	500	
Material	AISI 8620RH	
Young’s modulus/GPa	*E*_1_ = *E*_2_ = 210	
Poisson’s ratio *ν*	*ν*_1_ = *ν*_2_ = 0.3	
Yield strength *Y*/MPa	1300	

**Table 2:** Parameters of the genetic algorithm.

Population size, *N*	200
Maximum generation number, *G*_max_	500
Maximum stall generation number, *G*_s_	20
Function tolerance, *e*	10^−6^
Crossover fraction, *P*_c_	0.8

## Data Availability

The data presented in this study are available on request from the corresponding author.

## Nomenclature

critical plane	The plane that has the maximum shear strain range over a loading cycle
FSDP	Fatemi–Socie damage parameter
RS	Residual stress
*D*_FS_	The maximum FSDP on all the critical planes of a material point
(*D*_FS_)_max_	The maximum *D*_FS_ of all material points
*N*_f_	Fatigue initiation life of a material point
(*N*_f_)_min_	Minimum fatigue initiation life of all material points or fatigue initiation life of the gear surface
*α*	Plane orientation
*α*_c_	The orientation of the critical plane corresponding to *D*_FS_
Δ*γ*	The maximum shear strain range on a plane over a loading cycle
*σ*_n,max_	Maximum normal stress on a plane over a cycle
*σ*_surface_	RS at the surface
*σ*_max_	Peak RS
*y*_max_	Depth of *σ*_max_
*y*_core_	Depth where RS vanishes
*σ*_max,t_	The transition value for *σ*_max_
Subscripts	
opt	Values associated with the optimum RS distribution
1, 2, 3, …	Values associated with plane 1, 2, 3, …

## Appendix A

Hills et al. [28] provided the analytical solution for the stress field in the half-space subjected to Hertzian loading with shear traction, as shown in Figure 3b. At the surface,

(A1) $$\frac{\sigma_{x}}{p_{m}}=\left\{ \begin{array}{l} -\left[{\left(1-{x^{\prime }}^{2}\right)}^{0.5}+2\mu x^{\prime }\right],x^{\prime }\leq 1\\ -\mu \left[x^{\prime }-{\left({x^{\prime }}^{2}-1\right)}^{0.5}\right],x^{\prime }>1 \end{array}\right.$$

(A2) $$\frac{\sigma_{y}}{p_{m}}=\{\begin{array}{c} -{\left(1-{x^{\prime }}^{2}\right)}^{0.5},x^{\prime }\leq 1\\ 0,x^{\prime }>1 \end{array}$$

(A3) $$\frac{\tau_{xy}}{p_{m}}=\{\begin{array}{c} -\mu {\left(1-{x^{\prime }}^{2}\right)}^{0.5},x^{\prime }\leq 1\\ 0,x^{\prime }>1 \end{array}$$

where *p_m_* = (4*E*^*^*P*/(*πR*))^0.5^ is the maximum Hertizian pressure (see Equation (3)) and *x*′ = (*x* − *x*_c_)/*b*. In the subsurface,

(A4) $$\frac{\sigma_{x}}{p_{m}}=y^{\prime }\left[2-\frac{s}{{\left(1+s^{2}\right)}^{0.5}}-\frac{{\left(1+s^{2}\right)}^{0.5}}{s}-\frac{{x^{\prime }}^{2}s^{3}}{{\left(1+s^{2}\right)}^{1.5}\left(s^{4}+{y^{\prime }}^{2}\right)}\right]+\mu \left\{\frac{x^{\prime }{y^{\prime }}^{2}s}{{\left(1+s^{2}\right)}^{0.5}\left(s^{4}+{y^{\prime }}^{2}\right)}-2x^{\prime }\left[1-\frac{s}{{\left(1+s^{2}\right)}^{0.5}}\right]\right\}$$

(A5) $$\frac{\sigma_{y}}{p_{m}}=-\frac{{y^{\prime }}^{3}{\left(1+s^{2}\right)}^{0.5}}{s\left(s^{4}+{y^{\prime }}^{2}\right)}-\mu \left[\frac{x^{\prime }{y^{\prime }}^{2}s}{{\left(1+s^{2}\right)}^{0.5}\left(s^{4}+{y^{\prime }}^{2}\right)}\right]$$

(A6) $$\frac{\tau_{xy}}{p_{m}}=-\frac{x^{\prime }{y^{\prime }}^{2}s}{{\left(1+s^{2}\right)}^{0.5}\left(s^{4}+{y^{\prime }}^{2}\right)}+\mu y^{\prime }\left[2-\frac{s}{{\left(1+s^{2}\right)}^{0.5}}-\frac{{\left(1+s^{2}\right)}^{0.5}}{s}-\frac{{x^{\prime }}^{2}s^{3}}{{\left(1+s^{2}\right)}^{1.5}\left(s^{4}+{y^{\prime }}^{2}\right)}\right]$$

where *y*′ = *y*/*b* and *s* is a function of *x*′ and *y*′.

(A7) $$s^{2}=\frac{1}{2}\left\{{\left[{\left(1{x^{\prime }}^{2}-{y^{\prime }}^{2}\right)}^{2}+4{y^{\prime }}^{2}\right]}^{0.5}-\left(1-{x^{\prime }}^{2}-{y^{\prime }}^{2}\right)\right\}$$

The strain field can be obtained using Equations (A1)–(A7) and Hooke’s law.
